# Increased Sustainability of Carbon Dioxide Mineral Sequestration by a Technology Involving Fly Ash Stabilization

**DOI:** 10.3390/ma12172714

**Published:** 2019-08-24

**Authors:** Ahmad Assi, Stefania Federici, Fabjola Bilo, Annalisa Zacco, Laura E. Depero, Elza Bontempi

**Affiliations:** INSTM and Chemistry for Technologies Laboratory, Department of Mechanical and Industrial Engineering, University of Brescia, Via Branze, 38, 25123 Brescia, Italy

**Keywords:** fly ash, stabilization, municipal solid waste incineration, carbon dioxide sequestration

## Abstract

Mineral carbonation, involving reactions of alkaline earth oxides with CO_2,_ has received great attention, as a potential carbon dioxide sequestration technology. Indeed, once converted into mineral carbonate, CO_2_ can be permanently stored in an inert phase. Several studies have been focalized to the utilization of industrial waste as a feedstock and the reuse of some by-products as possible materials for the carbonation reactions. In this work municipal solid waste incineration fly ash and other ashes, as bottom ash, coal fly ash, flue gas desulphurization residues, and silica fume, are stabilized by low-cost technologies. In this context, the CO_2_ is used as a raw material to favor the chemical stabilization of the wastes, by taking advantage of the pH reduction. Four different stabilization treatments at room temperature are performed and the carbonation reaction evaluated for three months. The crystalline calcium carbonate phase was quantified by the Rietveld analysis of X-ray diffraction (XRD) patterns. Results highlight that the proposed stabilization strategy promotes CO_2_ sequestration, with the formation of different calcium carbonate phases, depending on the wastes. This new sustainable and promising technology can be an alternative to more onerous mineral carbonation processes for the carbon dioxide sequestration.

## 1. Introduction

Global carbon dioxide (CO_2_) emissions have continued to increase worldwide in the last decades, mainly due to the fossil fuels combustion [1]. Atmospheric CO_2_ concentration was above 400 ppm in 2016 and increased to 406.42 ppm in February 2017 [2]. Because of the fossil fuels abundance, industries are expected to shift to more sustainable fuel sources in several years. For this reason it is mandatory to experiment ways to reduce concentration of CO_2_ in the atmosphere [3]. Several methods for CO_2_ reduction (capturing and storing) have been proposed, including physical and chemical sequestration methods such as storage in geological formations, oceans, below the sea bed,; and mineral carbonation [4].

The mineral CO_2_ sequestration is today considered one of the promising method for reducing CO_2_ emissions [5]. The reactions involved in CO_2_ mineral carbonation consist in the mimic the process of natural rock weathering, where carbonic acid from the dissolution of CO_2_ in water is neutralized by alkaline metal minerals to form stable carbonate minerals [6,7,8]. Due to the stability of formed phases, the process does not require post-storage monitoring [9].

However, the slow reaction rate, material and energy requirements, and associated environmental issues created barriers to the technology implementation [10].

Then recently potential alternative to mineral carbonation with natural minerals was found in the use of industry-specific carbon dioxide sequestration methods, in which the CO_2_ emissions from industrial processes are offset by sequestration using by-products of these same processes [11], as for example steel-making slag [12], cement kiln dust [6], and mining wastes [13]. Also in the building industry, attention was paid to develop new cementitious construction materials (for example alkaline mortars, based on waste and fly ash), able not only to carbon dioxide sequestration, but also to accelerate curing and strength development of those materials [14,15].

Many processes that result in high CO_2_ emissions (as for example involving incineration) also produce considerable amount of solid alkaline waste products, such as combustion residues, ashes, and grinding residues [16]. In recent years, the literature attention was focalized on municipal solid waste incineration (MSWI) by-products; around 25%–30% bottom ash (BA, or MSWI BA) and 1%–3% fly ash (FA, or MSWI FA) of waste incinerated are generated in MSWI plants. MSWI BA mainly contains metals with low volatilization, while more volatile metals (such as Zn, Ni, Cu, As, Hg, Cr, and Cd) are found in MSWI FA generally in a leachable form [17,18].

As a consequence, BA residues of incineration are generally considered a by-product that can be reused, for example, in substitution of quartz sand in cement production. On the contrary, MSWI FA is managed as a toxic waste, and generally destined to landfill [17,19].

Considering that the processes generating FA emit a great amount of CO_2_, a sustainable approach to limit carbon dioxide emissions from MSWI FA can involve the waste use as feedstock for CO_2_ capture and storage. This principle is the basis of the “Azure chemistry” approach [20], that forecasts to restore the ecosystems by sustainable solutions in terms of energy, materials and emissions. Concerning the CO_2_ sequestration this approach requires low-energy paths, manufacturing and technologies reducing the use of non-renewable resources, and in which wastes and by-products are employed [21,22]. Our approach is following the Azure chemistry principle; the mineral carbonation made by wastes is readily achieved to store CO_2_ and will be a permanent solution compared to geological and ocean storage [9]. In addition the FA composition, containing unstable carbonated (or bi-carbonated) can reduce the CO_2_. Another reason is due to the waste geochemical instability and reactivity, derived from the fact that FA is generally formed at very high temperature and subsequently obtained by rapid cooling [23]. Finally, although CO_2_ reduction can be achieved by using FA in different methods, the primary technology is based on mineral carbonation, due to high content of alkali metal oxide [24,25], such as CaO.

This work focuses on the uses of MSWI FA for CO_2_ sequestration process, based on carbonation reactions occurring during the proposed stabilization process. In particular, this approach is innovative in respect to other proposed strategies for the following reasons:

a) The carbonation reactions are by-products of a stabilization procedure, based on the use of amorphous silica as leachable heavy metals stabilizer, that also involve carbon dioxide sequestration;
b) All the materials used in the process are wastes and by-products;
c) The reactions do not require control of temperature or pressure conditions. Indeed, it is fundamental to highlight that there are several studies focused on accelerated carbonation of MSW residues. In the majority of these works, accelerated carbonation tests were performed on humidified samples, applying pressures of CO_2_ and thermal treatments;
d) The process is realized in the frame of “Azure chemistry” approach [20]. Then its sustainability is guarantee by the approach fundamentals;

In this paper, the effects of carbonation reactions occurring by mixing MSWI FA, with Coal Fly Ash (CFA), Flue Gas Desulphurization (FGD) residues and silica fume are reported and discussed. Pulverized coal combustion produces CFA, that is a by-product in thermal power plants. FGD residues are produced as a by-product during the removal process of sulfur oxides in coal-burning power plants. This study demonstrates that chemical stabilization of MSWI FA using low-cost technology has the additional benefits to promote carbon dioxide sequestration.

This work is mainly based on X-ray diffraction (XRD) analysis, that allows to evaluate the changes of the crystalline phases, due to the carbonation reaction. It was recently shown how in-situ XRD facilities can be very useful to study carbonation [26]. Moreover, in this work, ex-situ XRD was performed, to evaluate amorphous quantification, by the addition of an internal standard.

The goal is to provide new scientific insights to CO_2_ reduction by the proposed technology by: (1) evaluating the effectiveness of the method to combine heavy metal immobilization and CO_2_ capture; (2) quantifying the amount of crystalline calcium carbonate that can be formed; (3) evaluating the effects of different industrial wastes and by-products to the sequestration; and (4) comparing the results obtained by using the proposed technologies in respect to literature data concerning MSWI FA carbonation.

## 2. Materials and Methods 

CFA and FGD residues were obtained by pulverized coal thermal power plant situated in Brescia, Italy. MSWI BA and FA were also kindly provided by A2A (Brescia, Italy). The tests were performed using MSWI FA and CFA [27]. Other ashes have been tested in combination or alternatively, such as FGD residues, BA (derived from the MSWI treatment), and silica fume. In Table 1, a description of sample preparation is reported. Then, about 200 mL of ultrapure de-ionized water (obtained from a Milli-Q purifier system (Millipore Direct Q TM, Burlington, MA, USA)) was added and the mixture was mixed for 20 min. All the samples were aged at room temperature for 3 months.

To verify the heavy metals immobilization, the leaching tests of Zn and Pb were performed according to the CEN normative (CEN EN 12457-2) [27,28]. 20 g of each specimen was mixed with 200 mL of Milli-Q water, with a ratio of 1:10 and mixed for 2 h at room temperature. 0.45 μm pore membranes were employed to filter the samples. The pH of the filtrates was measured by a pH meter (Metrohm, model 827 Lab). Leaching solutions were analyzed one (1 month) and two months (2 months) after the stabilization process in order to verify the efficacy of the process.

A S2 Picofox system from Bruker (Bruker AXS Microanalysis GmbH, Berlin, Germany) equipped with Mo tube operating at 50 kV and 750 µA and a Silicon Drift Detector (SDD) was employed for the elemental chemical analysis of the leachate solutions. A stock solution of 1 g/L Ga in nitric acid (Ga-ICP Standard Solution, Fluka, Sigma Aldrich, Saint Louis, MO, USA) was used as an internal standard in order to calculate the concentration of interested analytes present in the sample. Samples were prepared by weight. Approximately 0.010 g of 100 mg/L of Ga solution was added to the prepared solutions to obtain a final concentration of 1 mg/L Ga. Solutions were homogenized using a vortex shaker for 1 min at 2500 rpm. A 10 μL drop of sample was deposited in the center of a plexiglass reflector. Afterwards, the reflectors were dried on a hot plate at 50 °C under a laminar hood and the residues were measured. Three reflectors were prepared for each sample specimen and irradiated for 600 s of live time. TXRF spectra were analyzed with the instrumental software using routine deconvolution based on mono-element profiles to evaluate the peak areas.

For the crystalline calcium carbonate determination and the evaluation of the amorphous content, X-ray diffraction (XRD) measurements were performed by a PANalytical X’Pert PRO diffractometer equipped Cu Kα anode and operating at 40 kV and current of 40 mA. Scans (2θ) were performed from 5° to 90°, with a step interval of 0.0167°.

To identify the phase composition, the PANalytical X’Pert HighScore Plus version 2.1 (associated with the ICDD PDF2 database, 1998) was used. To evaluate the amount of the phases, the Rietveld analysis was performed by the PROFEX software packages (version 3.14.3, released March 17, 2019) [29].

The files used in the simulation were found in the Crystallography Open Database (COD) open access database [30]. Since Al_2_O_3_ (corundum) is not present, it was used as internal standard to quantify the amorphous phase.

XRD and Rietveld refinement were performed on samples to follow the carbonation process till to 3 months after the stabilization procedure.

## 3. Results

Chemical analysis of all materials used in the present paper, obtained from samples digestion, were already reported [31]. MSWI FA contains high amount of S, K, and Ca (>19 g/kg), while Fe is almost one order on magnitude lower than the value found in MSWI BA (about 4 g/kg). Indeed, Ca and Fe are the major elements in MSWI BA (>40 g/kg). K, S, P, and Cu are also found in relevant amounts (>6 g/kg). CFA mainly contains S, K, Ca, and Fe (>9 g/kg). FGD residues contain large amount of Ca (>160 g/kg) and S (>95 g/kg) [32].

**Figure 1:**
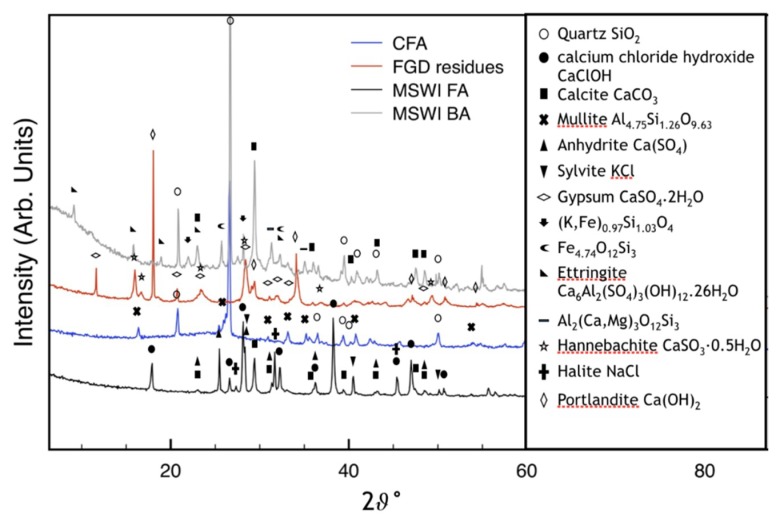
X-ray diffraction (XRD) patterns of MSWI FA, CFA, FGD residues, and MSWI BA.

Table 1 shows the XRD pattern collected on MSWI FA. By Rietveld analysis it was found that MSWI FA contains calcite (about 10%), CaClOH (about 24%), and soluble salts (NaCl, KCl, with total amount of about 9%). MSWI FA contains also a large amount of amorphous phase (about 56%). Calcite was attributed to the natural carbonation process, occurred during the MSWI FA storage [32]. Figure 1 shows also the XRD pattern of CFA, FGD residues and MSWI BA. In accord with previous reported data [33], the major crystalline phases of MSWI BA are ettringite, calcite, and quartz. Some peaks that can be ascribed to aluminum calcium (magnesium) silicate and potassium iron silicate phases are also detected. FGD residues contain portlandite, hannebachite, gypsum as the main crystalline phases. Finally, CFA contains quartz and mullite as the major crystalline phases.

Results of the leaching tests, obtained on MSWI FA, are shown in Table 2. Pb and Zn are found in high concentration in MSWI FA sample (35 and 9 mg/L, respectively), while concentrations of these elements in the other ashes result to be two order of magnitude lower. The high concentration of Zn and Pb in MSWI FA is likely related to the high pH (about 12) with consequent high Zn and Pb solubility.

The stabilization technology used is based on amorphous silica sources for metals immobilization [34]. Amorphous silica source can be found in Sample A, due to the silica fume addition, and in samples B and C, due to the use of MSWI BA [33]. Table 2 reports the concentration of heavy metals (Pb and Zn) detected in the leaching solutions of the stabilized materials, one and two months after the treatment.

All the stabilized samples revealed lower concentrations of Pb and Zn in their leaching solutions, in respect to the starting MSWI FA and the results are better after two months. Considering the pH of the leaching solutions, it results that it is always lowering with aging. This stabilization effect and the pH reduction were attributed to carbonation reactions, as diffusely discussed in several studies [32,35].

The main reactive species for carbon dioxide sequestration in MSWI FA are generally CaClOH (calcium chloride hydroxide) and Ca(OH)_2_ (portlandite).

CaClOH is available for carbonation reactions through the following reaction:

2CaOHCl + CO_2_ → CaCO_3_ + CaCl_2_ + H_2_O (1)

The carbonation reaction for Ca(OH)_2_ is:

Ca(OH)_2_ + CO_2_ → CaCO_3_ + H_2_O (2)

Indeed, natural carbonation of MSWI FA was generally regarded as an interesting process controlling pH variation and limiting the heavy metals solubility, but it is an extremely slow process, requiring decades [36].

The stabilization mechanism of the proposed technology, involving the mixing of different wastes and by-products, also involves carbonation reactions, that allow to reduce the pH of the stabilized materials and heavy metals leachability. Indeed, the reduction of heavy metals leaching was explained by two combined reactions, respectively due to the absorption in the amorphous silica and the carbonation. Moreover, the immobilization of residual heavy metals, after the stabilization technology obtained by lowering the pH (for example by forming insoluble phases), was attributed to indirect consequence of carbonation [32].

In present case, Ca(OH)_2_ cannot be found in the starting MSWI FA. However, this phase (portlandite) is available in FGD residues (see Figure 1). The XRD and Rietveld analysis were performed, collecting small portions of samples (about 1 g), at different aging times.

Rietveld refinement was performed because it is useful not only to made qualitative analysis of XRD patterns, since missing mineral phases inevitably involve significant differences between calculated and experimental patterns, but also because it allows to evaluate the amount of all detected phases. This was fundamental for our study to follow the evolution of the formed quantities of calcium carbonate crystalline phases, as a function of time and samples compositions. Moreover, the Rietveld method permits the quantification of the amorphous phase, since changes in the amorphous amount must be checked to obtain reliable information about phases evolution.

**Figure 2:**
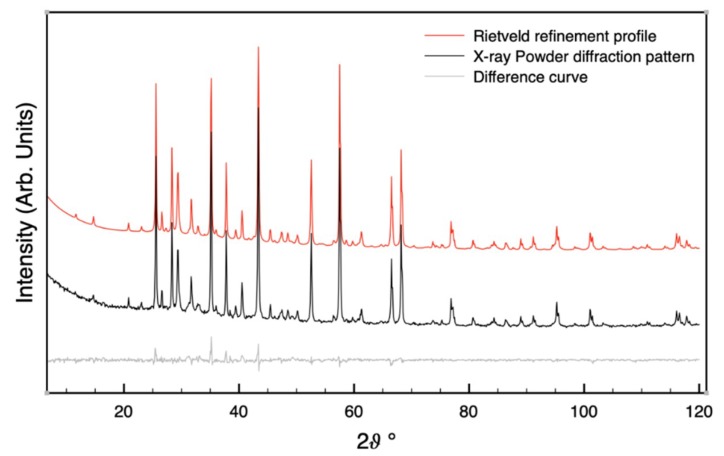
XRD pattern and corresponding Rietveld refined profile made on stabilized sample A, one month after the sample synthesis. Difference curve is also plotted.

Figure 2 shows the XRD pattern collected on stabilized sample A, one month after the sample synthesis. It also reports the results of the Rietveld refinement and the difference between experimental and calculated intensity is also plotted. A representative example is reported. The small differences obtained for the two patterns confirm the reliability of the qualitative characterization carried out.

Table 3 shows the results of the Rietveld analysis on all the samples, after different aging times. 

Following carbonation, the Ca(OH)_2_ and CaClOH peaks disappeared in the XRD patterns, whereas many CaCO_3_ peaks appeared. In particular, two carbonates were identified, calcite and aragonite, likely due to reactions (1) and (2). Calcite was already present in the MSWI FA possibly because of partial natural carbonation. Vaterite, the thermodynamically least stable phase among the three polymorphic forms of calcium carbonate, was detected in FA sample, as already found in similar experiments [32]. Vaterite is generally formed at a relatively low temperature condition (also at room temperature) [37].

**Figure 3:**
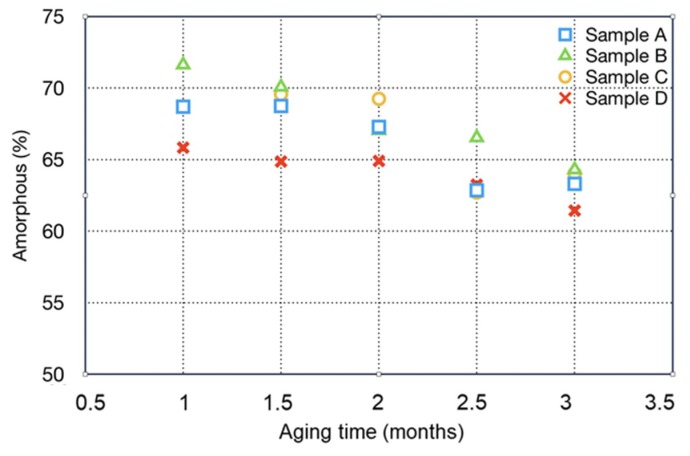
Amount of amorphous, evaluated by Rietveld method, as a function of samples aging time.

Another interesting result found by Rietveld analysis is the quantification of the amorphous phase and its variation during the investigated period. These data are reported in Figure 3. The amount of amorphous is quite high in all the samples (more than 65%) and decreases during reaction with the carbon dioxide. The reduction of the amorphous content is correlated with the increase of the crystalline phases. All the Rietveld results are reported in Table 3.

**Figure 4:**
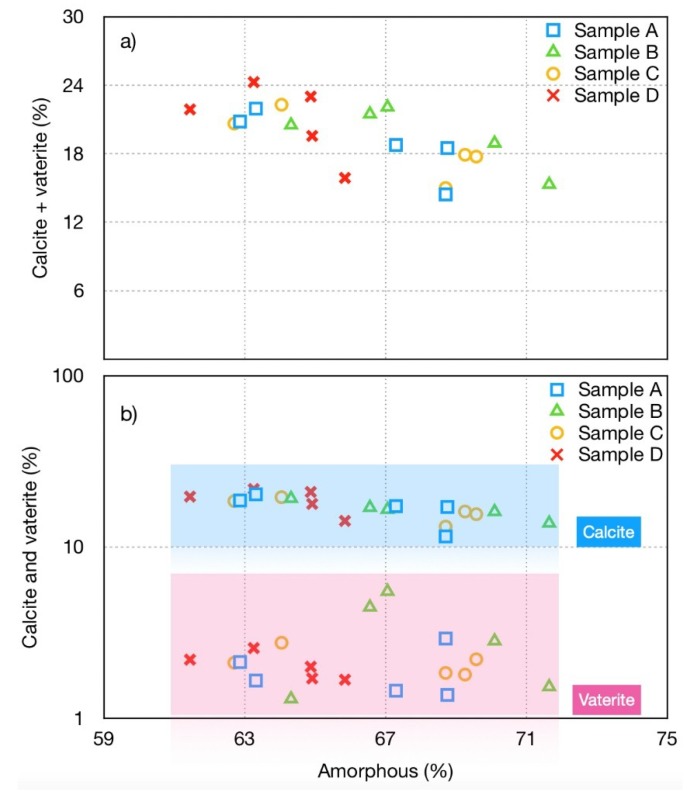
Amount of crystalline calcium carbonate phases (calcite and vaterite), evaluated as a global calcium carbonate crystalline phases (**a**) and separately (**b**). These data were calculated by Rietveld method, in respect to corresponding amorphous content.

Based on the amount of calcite and vaterite present in the original samples, the crystalline calcium carbonate content in the mixture induced by carbonation reactions was calculated. In Figure 4, it is shown that the amount of crystalline calcium carbonate phases (calcite and vaterite) increases when the amorphous phase decreases.

All the samples, before aging, contain calcite, in the range of 6%–10% (see Table 3) and vaterite was not detected. Considering the original sample, the CaCO_3_ content increased about 70% in samples A and D, while in sample C the CaCO_3_ is increased about 40% and in sample B it is about 30%. It is interesting to notice that in sample B, FGD (that contains portlandite) was not added. This may explain the low carbonation. Sample D, where the amount of FGD on total ashes content is the highest, shows the largest global increase of crystalline calcium carbonate, that was more than 70% in 3 months.

On this basis, we calculated that the treated material is able to sequestrate more than 90 g CO_2_/kg of ash (taking into account also the natural carbonation of MSWI FA). This result is great, because the maximum CO_2_ sequestration quantity reported for the carbonation of MSWI FA was about 58 g CO_2_/kg and the carbonation reactions were also supported by thermal annealing [38]. A very recent paper [39] reaches the maximum capacity of carbon dioxide sequestration of 60 g CO_2_/kg of FA at atmospheric pressure, but it was achieved at 600 °C with 20% H_2_O (g) addition.

The better results obtained in this work can likely be ascribed to the addition of FGD residues.

It is also important to highlight that amorphous calcium carbonate cannot be quantified by XRD and therefore, the amount of CO_2_ sequestrated is expected to be higher than that evaluated only considering the crystalline calcium carbonate.

Figure 4 shows the calcite + vaterite content to enlighten their behavior in respect to the change of the amorphous content. The data, plotted for the four different samples (in logarithm scale), show a linear inverse correlation between the amount of calcite and vaterite with the amorphous content. Moreover, sample B, which contains the highest amount of MSWI FA but not FGD residues, shows the largest amount of vaterite and the lowest amount of calcite. This result supports the hypothesis that the carbonation of FGD residues favors the calcite formation. On the contrary, the vaterite formation can be related to the MSWI FA carbonation (MSWI FA amount is 72% for sample B). 

It is expected that vaterite ultimately reverts to calcite [40]. Indeed, vaterite, that is less dense than calcite, can be formed by bio-mineralization, with a stability that can take several months. Some authors suggested that the formation of amorphous calcium carbonate is a transient intermediate, which transforms to the crystalline vaterite [40]. In Figure 3 it is shown that sample B has the highest reduction of the amorphous phase during the aging (more than 10% from month 1 to month 3), in respect to the other samples (that show generally a reduction of 7%). Then it is very likely that in this sample the amorphous calcium carbonate transforms in vaterite.

In Figure 3 is also shown that sample B, at the end of the aging, has a lower vaterite percentage (1.3%), in respect to 5.5% found two months after the treatment. This is in accordance with the possible phase transformation of vaterite into calcite, occurred as a stabilization of the metastable vaterite phase formed as a consequence of mineralization.

The amount of the other detected crystalline phases (gypsum, quartz, hannebachite, anhydrite, sylvite, and halite) was also evaluated (data are reported in Table 3) and no evident correlations with the change of the amorphous content amount were found. Thus, we can conclude that the increase of the crystalline calcium carbonate phases is due to crystallization of an amorphous phase. Indeed, the D sample, that shows the highest amount of crystalline CaCO_3_, has the lowest amount of amorphous percentage. 

## 4. Conclusions

The present study reports an experimental analysis of the potential application of a chemical stabilization technology, based on the use of wastes and by-products containing amorphous silica, to obtain carbon dioxide sequestration.

The results of leaching tests, made at different aging times, confirm its efficacy in terms of heavy metals immobilization. The occurring carbonation reactions are a positive by-product of the proposed technology. The XRD patterns of the four different stabilized samples provide information about the crystalline phase and in particular the percentage of crystalline calcium carbonate phases was obtained by Rietveld method. The amorphous content was also evaluated by using Al_2_O_3_ (corundum) as the internal standard. The changes during the aging of the calcite and vaterite content allow to enlighten the carbonation process. It was demonstrated that the carbonation of FGD residues favor the formation of calcite, while the vaterite formation can be due to MSWI FA carbonation. Finally, it was shown that in samples containing MSWI FA, the formation of vaterite from the amorphous phase also contributes to carbon dioxide sequestration. After the aging at room temperature, the metastable vaterite phase partially transforms in the more stable calcite. Results reveal that the treated materials are able to sequestrate till 90 g CO_2_/kg of ash.

In summary, we demonstrated that the stabilization procedure previously proposed [27] also significantly promotes the CO_2_ sequestration. Therefore, carbonation of MSWI FA obtained as a consequence of a stabilization technology could be a low-cost and promising technique for their treatment. For future activities, accelerated carbonation may be also studied with the aim to improve the velocity of the reactions and the amount of CO_2_ sequestrated

## 5. Patents

Patent number 102019000006651, 2019.

## Figures and Tables

**Table 1:** Samples description. MSWI FA: municipal solid waste incineration-fly ash; CFA: Coal Fly Ash; FGD: Flue Gas Desulphurization.

Samples	MSWI FA (g)	CFA (g)	FGD (g)	Silica Fume (g)	MSWI-BA (g)	%MSWI FA (%)	%FGD (%)
A	130	30	40	20	-	59.1	18.2
B	130	30	-	-	20	72.2	-
C	130	30	40	-	20	59.1	18.2
D	130	30	40	-	-	65	20

**Table 2:** Concentration of Zn and Pb in leaching solution of ashes and stabilized samples after one and two months. Results are reported as the average ± standard deviation of three TXRF measurements. Data about MSWI FA, BA, CFA, and FGD residues do not change in the two months.

Samples	pH	Months	Elemental Concentration (mg/L)					
			Zn			Pb		
MWSI-FA	12.18	-	8.80	±	4.30	34.60	±	2.40
MWSI-BA	10.69	-	0.05	±	0.04	0.09	±	0.05
CFA	11.81	-	0.24	±	0.02	0.13	±	0.03
FGD	12.68	-	0.1	±	0.04	<LOD		
A	11.73	1	0.15	±	0.09	<LOD		
	8.92	2	0.11	±	0.01	<LOD		
B	12.22	1	1.52	±	0.19	12.20	±	0.70
	10.23	2	0.14	±	0.00	<LOD		
C	12.06	1	0.39	±	0.02	3.20	±	0.50
	10.37	2	0.07	±	0.01	<LOD		
D	12.22	1	0.80	±	0.40	6.40	±	1.60
	11.07	2	0.07	±	0.001	<LOD		

<LOD—Limit Of Detection.

**Table 3:** Results of Rietveld analysis at different aging times (semi-quantitative analysis).

Samples	Months	Amorphous (%)	Calcite (%)	Hannebachite (%)	Thaumasite (%)	Gypsum (%)	Quartz (%)	Vaterite (%)	Sylvite (%)	Halite (%)	Anhydrite (%)
Sample A	0		6 *								
	1	69	12	5	<1	1	1	3	2	3	4
	1.5	69	17	4	<1	3	<1	2	1	2	2
	2	67	17	5	<1	<1	1	1	2	2	5
	2.5	63	19	5	<1	<1	1	2	1	3	5
	3	63	20	4	<1	3	2	2	1	2	3
											
Sample B	0		11*								
	1	72	14	2	<1	1	2	2	2	4	<1
	1.5	70	16	<1	3	1	2	3	1	2	<1
	2	67	17	<1	<1	1	3	6	<1	2	3
	2.5	67	17	<1	<1	1	2	4	1	2	4
	3	64	19	3	<1	2	<1	1	1	3	4
											
Sample C	0		9*								
	1	69	13	6	<1	1	<1	2	2	3	3
	1.5	70	16	5	<1	2	1	2	1	2	2
	2	69	16	4	<1	2	1	2	1	1	3
	2.5	63	19	4	<1	1	2	2	1	2	6
	3	64.	20	3	<1	1	<1	3	1	1	5
											
Sample D	0		7*								
	1	66	14	7	<1	<1	<1	2	2.	3	4
	1.5	65	21	6	<1	1	1	2	1	2	1
	2	65	18	6	<1	2	1	2	<1	2	3
	2.5	63	22	5	<1	<1	2	3	<1	2	2
	3	61	20	4	<1	2	3	2	1	2	5

* indicates the amount of calcite present in the mixed powders, before reaction (month 0). It represents the natural carbonation contribution.
